# Giant viable hydatid cyst of the lung: a case report

**DOI:** 10.1186/1752-1947-2-359

**Published:** 2008-11-25

**Authors:** Nagi Homesh Ghallab, Ali Ali Alsabahi

**Affiliations:** 1 Surgical Department Sana'a University and El-thawra Teaching Hospital, Sana'a, Yemen

## Abstract

**Introduction:**

Hydatid disease is a parasitic infestation caused by *Echinococcus granulosus*. The resulting large cysts in the lung are a special clinical entity called giant hydatid cysts.

**Case presentation:**

An 18-year-old Yemeni woman presented with a dry cough and mild fever, with no history of chest pain, dyspnoea or weight loss. Chest X-ray revealed a homogenous opacity almost replacing the right lung. The patient underwent surgery which revealed a large, viable hydatid cyst measuring 26 × 18 × 5 cm.

**Conclusion:**

This case report provides evidence that non-complicated hydatid cysts, even if very large, have a good prognosis and can be safely treated by parenchyma-preserving surgery.

## Introduction

Hydatid disease is a parasitic infestation caused by *Echinococcus Granulosus *characterized by cystic lesions in the liver and lungs but rarely in other parts of the body [1,2]. Giant hydatid cysts of the lung are defined as cysts measuring 10 cm or more [3]. In our Institute we have been treating giant hydatid cysts of the lung for 15 years, but never more than 20 cm in diameter and most of them were complicated and non-viable.

## Case presentation

An 18-year-old Yemeni woman presented at the Otolaryngology clinic with a history of dry cough, sore throat and mild fever. She was diagnosed with upper airway infection and she confirmed that she had had similar attacks in the previous 3 years. A chest X-ray was ordered to exclude chronic chest infection. Surprisingly, the X-ray revealed nearly complete replacement of the right hemithorax with a dense homogenous opacity.

**Figure 1:**
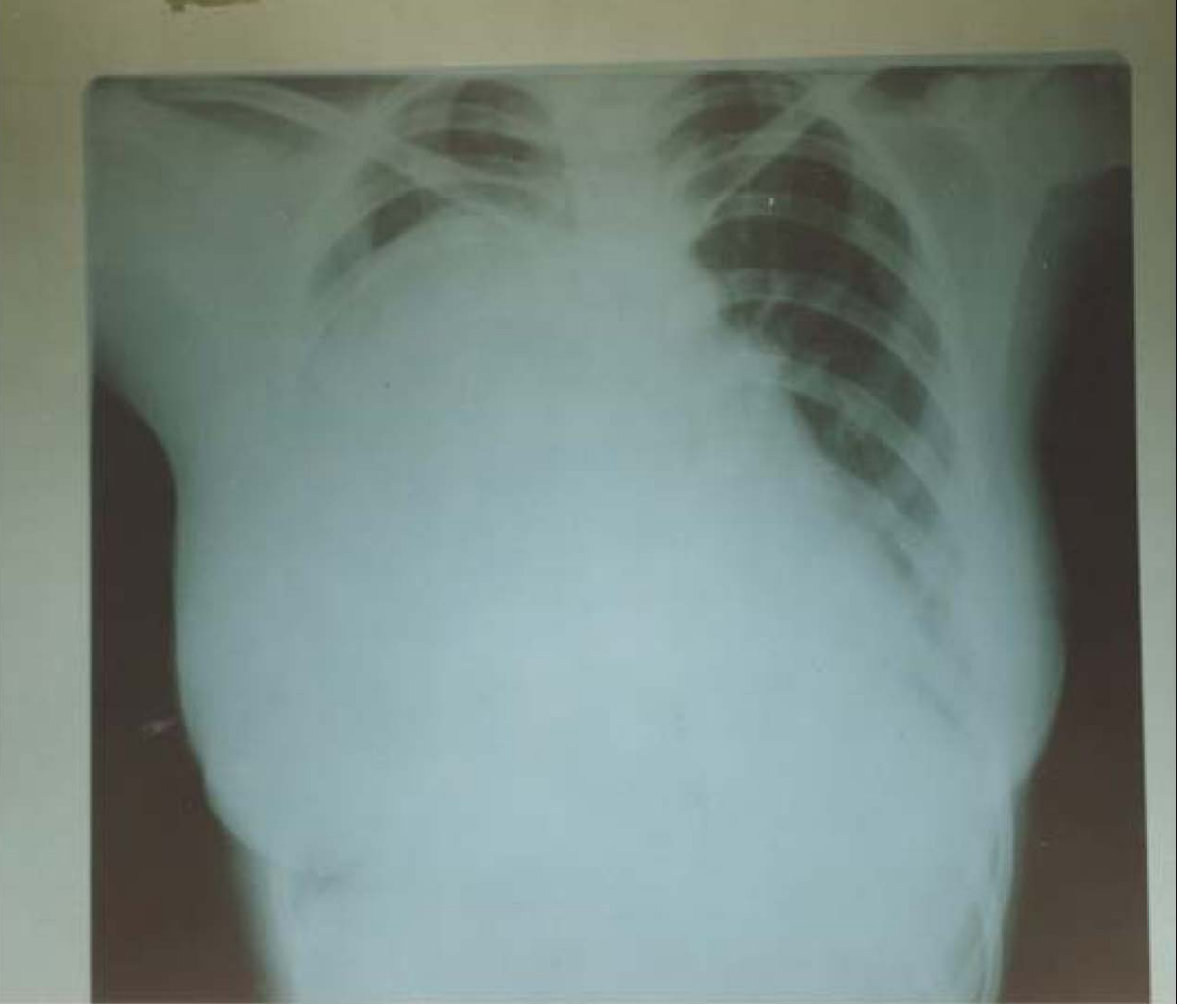
Chest X-ray showing a dense homogenous radiopaque opacity involving most of the right hemithorax.

The patient was then referred to the surgical clinic. Additional clinical imaging showed an impaired percussion note and diminished air entry over the right hemithorax. The chest X-ray was repeated and showed a very large, dense homogenous opacity occupying nearly 90% of the right lung (Figure 1). Due to the endemicity of hydatid disease in Yemen, our preliminary initial diagnosis was *Echinococcus *of the lung. After a week of preparatory albendazole treatment, the patient underwent parenchyma-preserving surgery. After right thoracotomy, the endocyst was enucleated intact with no spillage of the fluid; the bronchiolar communications were then sutured using 3/0 proline and capitonage; finally, the edges of the cyst were trimmed and sutured.

**Figure 2:**
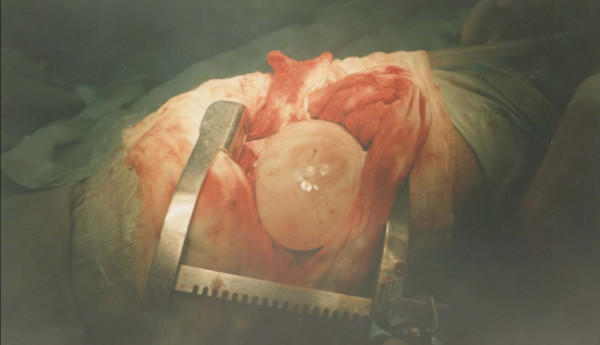
Right thoracotomy incision showing a very large white cyst delivered from the right lung, surrounded by gauze pads soaked with hypertonic saline.

**Figure 3:**
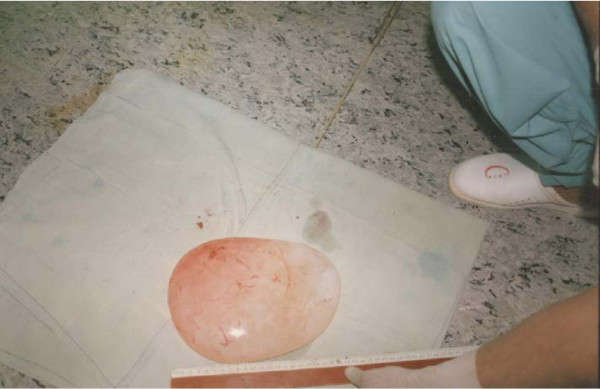
The delivered, very large, lung white cyst (giant hydatid cyst) with the greatest diameter measuring 26 cm.

The operation revealed a very large viable hydatid cyst measuring about 26 × 18 × 5 cm and containing more than 2 litres of fluid (Figures 2 and 3). The analysis of the fluid revealed viable scoleces. The postoperative course was uneventful and she was discharged after 7 days with a 4-week course of postoperative albendazole. The progress of patient follow-up was smooth.

## Discussion

Hydatid disease is a parasitic infestation caused by *Echinococus Granulosus *[1,2]. It is endemic in many countries and Yemen is one of the endemic regions [4]. The lungs are the second most common sites for hydatid cysts after the liver [1,2]. The majority of lung hydatid cysts are silent and either small or medium in size. Non-complicated hydatid cysts are usually discovered incidentally during routine chest X-rays for complaints other than chest diseases [5]. Giant hydatid cysts and complicated cysts, on the other hand, are usually symptomatic [6]. The common presentations are compression symptoms such as a dry cough in cases of very large cysts; a productive cough in cases associated with communication with the bronchial tree; and chest pain and dyspnoea in the case of rupture to the pleural cavity [6]. Anaphylactic shock is a rare presentation (seen in cases of rupture to the pleural cavity). The diagnosis is easy in endemic areas. The patient is usually in good general health in cases of non-complicated cysts and chest X-ray will show a well-circumscribed dense homogenous opacity [7]. A water-lily radiological sign is a diagnostic feature for a cyst associated with communication with small bronchioles and with a detached laminated membrane [7]. Productive cough of grape skin-like material is diagnostic in ruptured hydatid cysts communicated with medium sized bronchioles [7]. Some complicated cysts represent diagnostic challenges and to obtain a final diagnosis may require operative intervention [7].

In our case, the diagnosis was incidental when the patient had a chest X-ray that revealed a large, dense opacity occupying about 90% of the right hemithorax (Figure 1). Asymptomatic lesions in endemic areas should raise the threshold for the diagnosis of hydatid cysts of the lung.

The operative findings showed the whitish laminated membrane (Figures 2 and 3) indicative of hydatid cysts. Halezeroglu et al. [8] state that the large size of hydatid cysts and delayed diagnosis in younger age groups may correlate with higher lung-tissue elasticity and delayed symptoms. Hydatid cysts of the lung in our institute are usually treated medically (albendazole with a dose of 10 mg per kg of body weight for three courses of 28 days each, with a rest of 2 weeks in between) [4]. This medical treatment is effective for most small cysts where surgical intervention is not mandatory. Galanakis et al. [9] suggest that medical treatment alone can be sufficient for small pulmonary hydatid cysts. Larger cysts usually need surgical intervention in addition to albendazole (either pre-operative or pre- and post-operative). The appropriate surgical intervention in a large but non-complicated hydatid cyst is parenchyma-preserving surgery and includes cystotomy or cystotomy with capitonage, in addition to meticulous suturing of the communicating bronchioles [10]. Complicated hydatid cyst treatment consists of surgically and post-operatively administered albendazole only if daughter cysts are detected during the operation. This is in agreement with many other studies [4,5,9] recommending the administration of albendazole alone or in association with surgical treatment.

## Conclusion

Our conclusion is that non-complicated hydatid cysts have a good prognosis regardless of their size and can be safely treated by parenchyma-preserving surgery.

## Consent

Written informed consent was obtained from the patient for publication of this case report and accompanying images. A copy of the written consent is available for review by the Editor-in-Chief of this journal.

## Competing interests

The authors declare that they have no competing interests.

## Authors' contributions

NH and AA performed the operation and wrote the manuscript. Both authors read and approved the final manuscript.
